# Identification of patients for clinical risk assessment by prediction of cardiovascular risk using default risk factor values

**DOI:** 10.1186/1471-2458-8-25

**Published:** 2008-01-23

**Authors:** Tom Marshall

**Affiliations:** 1 Department of Public Health & Epidemiology, University of Birmingham, Edgbaston, Birmingham, B15 2TT, UK

## Abstract

**Background:**

To identify high risk patients without cardiovascular disease requires assessment of risk factors. Primary care providers must therefore determine which patients without cardiovascular disease should be highest priority for cardiovascular risk assessment. One approach is to prioritise patients for assessment using a prior estimate of their cardiovascular risk. This prior estimate of cardiovascular risk is derived from risk factor data that are routinely held in electronic medical records, with unknown blood pressure and cholesterol levels replaced by default values derived from national survey data. This paper analyses the test characteristics of using such a strategy for identification of high risk patients.

**Methods:**

Prior estimates of Framingham cardiovascular risk were derived in a population obtained from the Health Survey for England 2003. Receiver operating characteristics curves were constructed for using a prior estimate of cardiovascular risk to identify patients at greater than 20% ten-year cardiovascular risk. This was compared to strategies using age, or diabetic and antihypertensive treatment status to identify high risk patients.

**Results:**

The area under the curve for a prior estimate of cardiovascular risk calculated using minimum data (0.933, 95% CI: 0.925 to 0.941) is significantly greater than for a selection strategy based on age (0.892, 95% CI: 0.882 to 0.902), or diabetic and hypertensive status (0.608, 95% CI: 0.584 to 0.632).

**Conclusion:**

Using routine data held on primary care databases it is possible to identify a population at high risk of cardiovascular disease. Information technology to help primary care prioritise patients for cardiovascular disease prevention may improve the efficiency of cardiovascular risk assessment.

## Background

Because they are at high risk of cardiovascular events, patients with cardiovascular disease are the highest priority for preventive interventions. Some patients without cardiovascular disease are also at high risk and are the next priority for prevention. In patients without cardiovascular disease, the Framingham cardiovascular equation is widely used to determine probability of a cardiovascular event [1]. UK guidelines recommend treatment at a ten-year Framingham cardiovascular (CVD) risk of 20% [2]. Calculating Framingham cardiovascular risk requires knowledge of a patient's age, sex, diabetic status, smoking status, total cholesterol, HDL cholesterol and whether or not they have existing cardiovascular disease. Risk factor assessment requires patient time, staff time and laboratory tests. Furthermore, not all patients assessed are eligible for treatment. To make best use of resources for identification of patients eligible for preventive treatments it would be helpful to pre-select and prioritise for assessment those patients most likely to benefit from treatment. How could we do this before a patient's risk factors are known? In health systems where patients have electronic medical records it is possible to calculate all patients' cardiovascular risks before they attend for cardiovascular risk assessment. This is done using all the cardiovascular risk factors that are already recorded in the electronic medical records (such as age, sex and diabetic status) and using prior estimates of (default) cardiovascular risk factor values for risk factor values that are unknown. Calculating a prior estimate of cardiovascular risk allows primary care providers to prioritise assessment of patients whose cardiovascular risks are highest and who are therefore most likely to be eligible for and benefit from assessment.

The cost effectiveness of a preventive strategy using this approach has previously been described [3] and the approach has been recommended in the recent draft NICE guidelines for lipid lowering [4]. To facilitate this strategy, software has been produced by iSoft [iSOFT, Daventry Road, Banbury, Oxfordshire, OX16 3JT.] and MSDi [MSDi, Hertford Road, Hoddesdon, Hertfordshire, EN11 9BU.] to calculate prior estimates of cardiovascular risk. However, to date details of the method for calculating prior estimates of cardiovascular risk have not been published.

This paper describes how default risk factor values may be obtained and used to calculate prior estimates of cardiovascular risk. Since diagnostic algorithms work well in the populations from which they are derived, they should be validated in a different data set. The paper validates the prior estimates of cardiovascular risk by using them to categorise patients in a separate population as high-risk.

## Methods

Electronic medical records include age, sex and generally include accurate information on antihypertensive drug treatment status and diabetic status. However for some patients no additional risk factor information may be available. In order to calculate cardiovascular risk, default risk factor values therefore may need to be provided for smoking status, total cholesterol, HDL cholesterol and blood pressure. Default risk factor values are the most likely values for the patient.

At all ages and in both sexes, non-smokers, non-diabetics, those without cardiovascular disease and those not on antihypertensive treatment outnumber smokers, diabetics, those with cardiovascular disease and those on antihypertensive treatment. For each of these risk factors therefore the default is that the risk factor is absent.

For continuous variables (total cholesterol, HDL cholesterol, systolic blood pressure), default risk factor values were derived from the Health Survey for England of 1998 [5]. This was done as follows. The survey population was divided into eight age bands (16–24, 35–34, 35,44, 45–54, 55–64, 65–74, 75–84 and 85+) and two gender groups (male and female). These sixteen groups were subdivided into those taking and not taking antihypertensive treatment; those with and without cardiovascular disease; those with and without diabetes; smokers and non-smokers. This made a total of 256 categories. For each of these categories, average cholesterol level, average high-density lipoprotein cholesterol (HDL) level, average systolic blood pressure and average diastolic blood pressure were calculated.

Some categories (e.g. males, 16–24, on antihypertensive treatment, with cardiovascular disease, with diabetes and smokers) are very uncommon. Because of this it is not possible to calculate a stable average for these categories. Where the Health Survey for England 1998 contained fewer than 10 individuals in a category, smokers were merged with non-smokers. If the category still contains fewer than 10 individuals, diabetics were merged with non-diabetics. If the category still contains fewer than 10 individuals, those with and without cardiovascular disease were merged. If the category still contains fewer than 10 individuals, those taking and not taking antihypertensive treatment were merged. In this way a list of default blood pressures was calculated for every possible age, sex and risk factor category. The values are available on-line [6].

The diagnostic value of CVD risk estimates derived using default risk factor values was investigated by testing the model in the Health Survey for England of 2003 [5]. Because the Framingham risk equation was derived from individuals aged 30 to 74 without cardiovascular disease, the population used for validation was all patients in this age group without cardiovascular disease. Patients on antihypertensive treatment and patients with diabetes were included in the validation population. There are 18,553 individuals in the Health Survey for England 2003: 10,741 are aged 30 to 74, of these 4,954 of these have sufficient cardiovascular risk factor information recorded to calculate CVD risk. This 4,954 includes 120 individuals who have cardiovascular disease. Excluding them leaves 4,834 individuals aged 30 to 74, free from cardiovascular disease but with sufficient risk factors to calculate CVD risk.

In England, a population of 12,500 would be expected to include about this number of individuals aged 30 to 74 without cardiovascular disease. This is roughly equivalent to the population cared for by a large group practice. The risk factor data were entered into SPSS and ten-year cardiovascular risks were each calculated for each individual – their "true" cardiovascular risk.

The reference standard for cardiovascular risk assessment is full clinical risk factor assessment. This means assessment of all cardiovascular risk factors, using the mean of blood pressures taken at two clinic visits and a single measure of total cholesterol and HDL cholesterol. Variation in measured risk factors can have a significant impact on the identification of patients as eligible for treatment, it is therefore important to incorporate this effect into the model [7-10]. In order to model this, a "clinically determined" cardiovascular risk was calculated for each individual, based on the mean of two clinically measured blood pressures and cholesterol levels [1]. This was added to the dataset. Clinically measured blood pressure incorporates the effects of chance (biological) variation on blood pressure and cholesterol measurement. Two measured blood pressures were generated for each individual in the population using a previously described methodology [7-9]. This method adjusts true blood pressure (the survey blood pressure) by an error term. [Measured BP = True BP × (1 + Error term)]. A series of normally distributed error terms are generated in Excel as random numbers with a mean of zero and a standard deviation equal to the coefficient of variation of between-visit, measured blood pressure. This between-visit coefficient of variation is derived from meta-analysis [11]. Two clinically measured cholesterol levels were also generated for each individual. Measured cholesterol levels incorporate an error term based on the coefficient of variation derived from published studies: 7.2% for total cholesterol and 7.5% for HDL cholesterol [12]. In effect, the clinically determined cardiovascular risk is an estimate of the true cardiovascular risk.

Electronic medical records almost always contain accurate data on age, gender, antihypertensive drug treatment status and diabetic status. If electronic medical records are available these are the minimum data that are available for estimation of cardiovascular risk. A "minimum data" estimate of cardiovascular risk was calculated using these data, with default risk factor values for missing information. Each patient was assigned to one the 256 risk factor categories and assigned default risk factor values appropriate to that category.

Electronic medical records often contain smoking status and an estimate of blood pressure. A "semi-complete data" estimate of cardiovascular risk was calculated with smoking status a single blood pressure in addition to minimum data.

There are other methods of prioritising patients for cardiovascular risk assessment. One approach is to prioritise diabetics and hypertensive patients (those already on antihypertensive treatment). This was recommended in the National Service Framework for Coronary Heart Disease [13]. Under such a strategy, hypertensive diabetics are the highest priority, followed by diabetics and hypertensive patients. Another approach is to prioritise patients by age, assessing the oldest first. For comparison, strategies prioritising patients using these methods are also assessed. The five modelled strategies are outlined in Table 1.

**Table 1:** Descriptions of the ten-year cardiovascular risks and prioritisation strategies used in this paper

Label for strategy	Risk factors	Strategy
Clinical CVD risk	Age, sex, diabetic status, antihypertensive treatment status, smoking status. Clinically estimated blood pressure (mean of two measurements), total cholesterol, and HDL cholesterol.	Highest risk first
Semi-complete data	Age, sex, diabetic status, antihypertensive treatment status, smoking status and clinically estimated blood pressure (one measurement)	Highest risk first
Minimum data	Age, sex, diabetic status, antihypertensive treatment status.	Highest risk first
NSF-CHD	Diabetic status, antihypertensive treatment status	Hypertensive diabetics, then diabetics, hypertensives & others
Age	Age	Oldest first

Receiver operating characteristic (ROC) curves illustrate the ability of a diagnostic test to discriminate, in this case between "true" ten-year cardiovascular risk greater and less than 20%. They plot the relationship between sensitivity and one minus specificity at a range of cut off test values. Taking true cardiovascular risk as the reference standard, ROC curves were constructed in SPSS 14.0 for "clinically estimated", semi-complete data" and "minimum data" risk estimates, for a prioritising diabetic and hypertensive patients and for a strategy prioritising by age. ROC curves are summarised by the area under the curve (C-statistic).

For each of the strategies the sensitivity and specificity of assessing the highest priority decile of the population aged 30 to 74 was calculated. This is intended to illustrate the effects of implementing patient identification strategies informed by prioritisation of patients at high risk of CVD in primary care.

## Results

**Figure 1:**
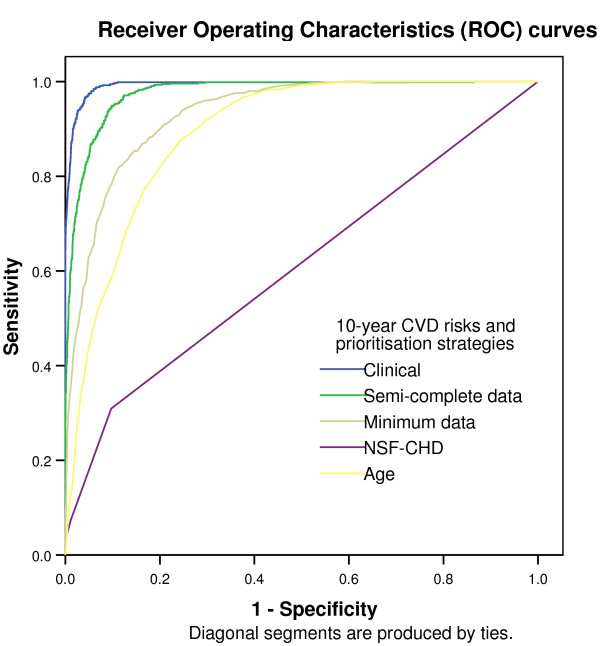
**Receiver operating characteristic curve of the ability of an estimate of cardiovascular risk to diagnose a true ten-year cardiovascular risk of over 20%**. The areas under the curves are shown in Table 2.

Seven hundred and fifty (15.5%) of the 4,834 patients are at greater than 20% ten-year cardiovascular risk. The ROC curves are shown in Figure 1.

Area under the curve is significantly greater for a strategy assessing patients in descending order of age (C-statistic 0.892, 95% CI: 0.882 to 0.902) than for a strategy prioritising diabetics and hypertensives (C-statistic 0.608, 95% CI: 0.584 to 0.632). A strategy prioritising patients for assessment by a prior estimate of cardiovascular risk based on minimum data (age, sex, diabetic and antihypertensive treatment status) has a significantly greater area under the curve (C-statistic 0.933, 95% CI: 0.925 to 0.941) than one prioritising by age. A strategy prioritising patients for assessment by a prior estimate of cardiovascular risk based on semi-complete data (age, sex, diabetic, antihypertensive treatment status, smoking status and a single blood pressure) has a significantly greater area under the curve (C-statistic 0.976, 95% CI: 0.972 to 0.980) than one prioritising by age. (Table 2)

**Table 2:** Areas under the curve for different methods of identifying patients at greater than 20% ten-year cardiovascular risk from a population of 4651 adults aged 30 to 74

Strategy	Area under the curve	Standard Error	Asymptotic 95% Confidence Interval
Clinical CVD risk	0.993	0.001	(0.991 – 0.996)
Semi-complete data	0.976	0.002	(0.972 – 0.980)
Minimum data	0.933	0.004	(0.925 – 0.941)
NSF-CHD	0.608	0.012	(0.584 – 0.632)
Age	0.892	0.005	(0.882 – 0.902)

Using semi-complete data to select 10% of persons aged 30 to 74 for CVD risk factor assessment has a sensitivity of 0.589, a specificity of 0.929 and a positive predictive value of 0.915. In other words, a practice following such a strategy can identify 58.9% of high-risk patients by assessing only one tenth of persons aged 30 to 74. Furthermore, 91.5% of those assessed will be at high risk. Using minimum data to select 10% of persons for assessment has a sensitivity of 0.511, a specificity of 0.915 and a positive predictive value of 0.754. Using only age to select 10% of persons for assessment has a sensitivity of 0.425, a specificity of 0.900 and a positive predictive value of 0.624. Using the National Service Framework strategy to select 10% of persons for assessment has a sensitivity of 0.253, a specificity of 0.871 and a positive predictive value of 0.393.

## Discussion

The principal problem with ROC curves is that they may be based on biased populations. However a population drawn from the Health Survey for England is unlikely to be biased. A second problem is that the analysis is carried out on a large population. This may make clinically trivial differences between the receiver operating characteristics of different strategies statistically significant. There are only small differences between using prior risk estimation in a practice with only age, sex, diabetic status and antihypertensive drug treatment status on all patients and one that also has smoking status and a blood pressure on all patients. Nevertheless it is clear that the strategies prioritising patients by any prior estimate of cardiovascular risk are a significant improvement on using age, or diabetic and hypertensive status.

This paper demonstrates that a prior estimate of cardiovascular risk based on data commonly held in electronic medical records has valuable characteristics as a screening test for high risk of cardiovascular disease. This is not to suggest that it is a substitute for cardiovascular risk assessment, rather that it is useful for prioritising patients for such an assessment. A cardiovascular assessment strategy based on a prior estimate of cardiovascular risk is clearly superior to prioritising diabetics and hypertensives or prioritising by age. Using such an approach, it is possible to identify a population of whom the majority are at high risk of cardiovascular disease. This population comprises the great majority of high-risk patients. Using information technology to calculate prior estimates of risk and rank patients by their estimated risk would greatly facilitate such a strategy. Such developments are now in place. Information technology can either provide electronic prompts to remind primary care physicians to assess patients opportunistically when they consult or can be used to produce lists of patients for active invitation and assessment.

In primary care, some patients have complete risk factor information while others have some information missing: most often, cholesterol levels [14]. The most efficient way to make use of this information is therefore to use recorded risk factor information when it is available and default values when it is not.

The test characteristics of the selection strategy depend on the number of patients identified for assessment. As more patients are identified for assessment a greater proportion of these identified patients are not high-risk at > 20% ten-year CVD risk. However, any selection strategy has a higher specificity than unselected assessment.

This paper describes pre-selection using prior estimates of cardiovascular risk using the Framingham cardiovascular risk equation in an English population. Other cardiovascular risk equations have been derived from different original data sources in continental Europe [15], Scotland [16] and the UK [17]. It is possible that future guidelines may adopt a different cardiovascular risk equation to determine eligibility for treatment. Calculating prior estimates of cardiovascular risk is not dependent on any single risk equation, it requires only that some of the principal determinants of cardiovascular risk are known and that default risk factor values can be obtained for the population to whom the equation is to be applied. It would be of interest to determine the receiver operating characteristics of different equations in different populations. The main determinants of cardiovascular risk (age, sex, smoking status, diabetic status, blood pressure, cholesterol levels) are the same in all risk equations. There is therefore no reason to believe that the findings would be fundamentally different in other populations.

## Conclusion

When identifying patients for primary prevention of cardiovascular disease, selecting patients for cardiovascular risk assessment using a prior estimate of cardiovascular risk is clearly a more efficient strategy than selecting based on other criteria. Software to assist in this process has the potential to improve the identification of patients at high risk of cardiovascular disease.

## Competing interests

The author(s) declare that they have no competing interests.

## Authors' contributions

Tom Marshall obtained the data, carried out the analysis and wrote the paper.

## Appendix

### What this paper adds

When blood pressure and cholesterol levels are unknown it is possible to use routine survey data to calculate a prior estimate of cardiovascular risk.

Using even the minimum of data available to primary care teams in their electronic medical records databases it therefore is possible to predict cardiovascular risk.

This risk prediction is sufficiently accurate to prioritise patients for cardiovascular disease assessment.

### Policy Implications

More use could be made of routine data that are held in electronic medical records databases in primary care. Information technology should be developed to make convert this database information into useful knowledge to guide cardiovascular prevention. Primary care teams should be encouraged to make use of such knowledge.

## Pre-publication history

The pre-publication history for this paper can be accessed here:


